# Development and Evaluation of a Miniaturized Taste Sensor Chip

**DOI:** 10.3390/s111009878

**Published:** 2011-10-20

**Authors:** Yusuke Tahara, Akihiro Ikeda, Yoshihiro Maehara, Masaaki Habara, Kiyoshi Toko

**Affiliations:** 1 Graduate School of Information Science and Electrical Engineering, Kyushu University, 744 Motooka, Nishi-ku, Fukuoka, 819-0395, Japan; E-Mails: ikeda@ed.kyushu-u.ac.jp (A.I.); maehara@belab.ed.kyushu-u.ac.jp (Y.M.); toko@belab.ed.kyushu-u.ac.jp (K.T.); 2 Intelligent Sensor Technology, Inc., 5-1-1 Onna, Atsugi-shi, Kanagawa 243-0032, Japan; E-Mail: Habara.Masaaki@insent.co.jp

**Keywords:** taste sensor, sensor chip, artificial lipid membrane, electrolyte layer

## Abstract

A miniaturized taste sensor chip was designed for use in a portable-type taste sensing system. The fabricated sensor chip (40 mm × 26 mm × 2.2 mm) has multiple taste-sensing sites consisting of a poly(hydroxyethyl methacrylate) hydrogel with KCl as the electrolyte layer for stability of the membrane potential and artificial lipid membranes as the taste sensing elements. The sensor responses to the standard taste substances showed high accuracy and good reproducibility, which is comparable with the performance of the sensor probe of the commercialized taste sensing system. Thus, the fabricated taste sensor chip could be used as a key element for the realization of a portable-type taste sensing system.

## 1. Introduction

**Figure 1.**
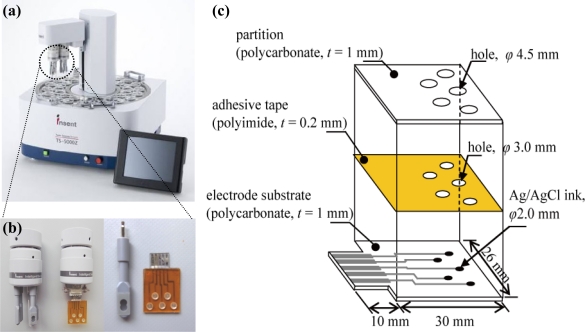
**(a)** External view of the commercialized taste sensing system. **(b)** Extended view of connecter parts with sensor probes and the sensor chip. **(c)** Structure of the fabricated sensor chip.

A taste sensor including an artificial lipid membrane that can evaluate various qualities of taste has been used in food production and quality-control applications. The sensor uses artificial lipid membranes as a recognition element, which transforms taste information generated by chemical substances into electric-potential change [1–4]. This taste detection system has been commercialized by Intelligent Sensor Technology, Inc., Japan [Figure 1(a)]. The taste sensing system consists of taste sensor probes, a controller and a data processing terminal. The sensor surface is composed of an attached artificial lipid membrane. The sensor probe is composed of a silver-wire electrode, the surface of which is coated with Ag/AgCl, with a sensor body made of polypropylene, and an artificial lipid membrane made by mixing lipids with a polymer. The internal cavity of the artificial lipid membrane sensor probe is filled with the internal solution. The membrane potential of the artificial lipid membrane is changed when the artificial lipid membrane reacts with a ‘taste substance’ in a sample solution [4]. The taste sensing system has been widely applied in manufacturing of beverages and production of foodstuffs such as green tea [5], milk [6], rice [7], soy sauce [8] and pork [9].

The commercialized sensing instrument is a laboratory-based system which is heavy and expensive. If a portable system could be developed, the taste sensor would be more widely used. In previous work, we have reported the development of a miniaturized taste sensor chip for the portable-type taste sensing system [10]. However, the precision, accuracy and reproducibility of the developed sensor chips need to be improved if compared with the commercialized taste sensor. A major problem for the miniaturized sensor chip is that the membrane potential is not stable when it is changed by taste substances.

Various types of miniaturized and integrated chemical sensors, such as Ion Selective Electrode [11], ISFET [12], and other ion sensors have been proposed. Some studies have demonstrated that the sensor signal from a miniaturized ion selective chip electrode can achieve high stability in terms of electric-potential by using hydrogel with KCl as the electrolyte layer [13,14]. In this study, we report an improved design for the miniaturized taste sensor chip based on the KCl hydrogel electrolyte layer. The performance of the five sensor chips for saltiness, sourness, umami, bitterness and astringency were compared with the conventional sensor probes by measuring the taste substances, and the measurement repeatability was evaluated.

## 2. Experimental Section

### 2.1. Reagents

Tetradodecylammonium chloride, trioctylmethylammonium chloride, and tetrahydrofuran were purchased from Sigma-Aldrich, Inc. (St Louis, MO, USA). *n*-Tetradecyl alcohol, potassium chloride (KCl), tartaric acid, monosodium glutamate (MSG), and tannic acid were purchased from Kanto Chemical Co., Inc. (Tokyo, Japan). Dioctyl phenylphoshonate and 2-nitrophenyl octyl ether were purchased from Dojindo Laboratories (Kumamoto, Japan). Phosphoric acid di(2-ethylhexyl) ester, trioctylmethylammonium chloride and oleic acid were purchased from Tokyo Chemical Industry Co., Ltd. (Tokyo, Japan). Polyvinyl chloride (PVC) was purchased from Wako Pure Chemical Industries, Ltd. (Osaka, Japan). Iso-alpha acid was purchased from Intelligent Sensor Technology Inc. (Kanagawa, Japan). All aqueous solutions were prepared in distilled water.

### 2.2. Taste Sensor Chip

A miniaturized sensor chip for the detection of taste substances was designed that consists of a Ti/Ag patterned electrode on a polycarbonate substrate, a polyimide double-faced adhesive tape (Kapton 4390, 3M) and a partition [Figure 1(c)]. The Ti/Ag patterning electrode was fabricated by using a standard photolithography technique. A Ti layer was deposited by rf plasma sputtering onto the polycarbonate substrate as an adhesive layer between the substrate and the Ag layer. Next, an Ag layer was deposited by following the same procedure. A photoresist layer was coated onto the substrate by the spin-coating method and then photolithography was performed to expose UV light through a photo-mask to define the electrode patterns. After development, the silver and titanium were etched in a mixed solution containing 10 wt% NH_3_ and 30 wt% H_2_O_2_, using the patterned resist as a mask. The substrate was washed in ethanol. Finally, Ag/AgCl ink (Ag/AgCl Ink for reference electrode, BAS, Japan) was applied to the electrode. To set up an electrolyte layer and an artificial lipid membrane, the polyimide double-faced adhesive tape and the partition were set up through holes of 3.0 mm and 4.5 mm in diameter, respectively.

### 2.3. Fabrication of Taste Sensing Sites

**Figure 2.**
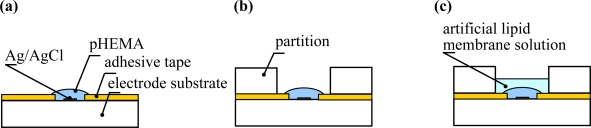
Manufacturing procedure for taste sensing site. **(a)** Forming the electrolyte layer. **(b)** Setting of the partition. **(c)** Deposition of artificial lipid membrane solution.

The taste sensing sites consisted of an electrolyte layer and an artificial lipid membrane. In order to form the electrolyte layer, polyimide double-faced adhesive tape was used to label the Ti/Ag patterned electrode. A poly(hydroxyethyl methacrylate) hydrogel (pHEMA) was polymerized by irradiation with UV light as the electrolyte layer. In the first step, 4 μL of a HEMA mixture solution consisting of 60 wt% hydroxyethyl methacrylate, 38 wt% ethylene glycol, 1 wt% dimethoxy-2-phenylacethophenone, and 1 wt% tetraethylene glycol dimethacrylate was dropped onto the electrode with the adhesive tape and was polymerized by UV light (2–3 mW/cm^2^) for 4 min [Figure 2(a)]. After polymerization, 1 mL of 1.7 M KCl solution was dropped onto it and the solution was removed after incubation for 10 hours. Twenty microliters of the artificial lipid membrane solution was deposited twice on the pHEMA layer of the sensing site with the partition, and then desiccated at room temperature for 10 hours [Figure 2(b,c)]. Finally, the sensor chip was conditioned for 1 day in a solution of 30 mM KCl and 0.3 mM tartaric acid before measurement.

### 2.4. Artificial Lipid Membrane

Five artificial lipid membrane solutions were prepared for the performance evaluation of the taste sensor chip and the sensor prove. The chemical component of the membrane (include lipids and plasticizers) are summarized in Table 1. The details on the preparation of the taste sensor probe can be referred from a previous report [3]. In this study, KCl, tartaric acid, MSG, iso-alpha acid and tannic acid were chosen as standard taste substances to represent saltiness, sourness, umami, bitterness and astringency, respectively.

### 2.5. Evaluation of the Sensor Chip

The responses of the fabricated sensor chips to each taste substance were measured and compared with the sensor probes. A solution consisting of 30 mM KCl and 0.3 mM tartaric acid was used as a reference solution. Different concentrations were prepared for each of these substances, as follows: saltiness substances; 37.5, 75, 150, 300 mM in 30 mM tartaric acid, sourness substances; 0.37, 0.75, 1.5, 3 mM tannic acid in 30 mM KCl, umami substances; 0.3, 0.6, 1.25, 2.5, 5, 10 mM MSG in reference solution, bitterness substances; 0.0006, 0.0012, 0.0025, 0.005, 0.01 vol% iso-alpha acid in a reference solution, astringency substances; 0.0016, 0.0031, 0.0063, 0.0125, 0.025, 0.05 wt% tannic acid in a reference solution. In order to assess the repeatability of the sensor, the fabricated sensor chips were used to measure the standard taste substances and the measurement with four duplicates were conducted once a day for 20 consecutive days.

### 2.6. Measurement Procedure

The measurements were performed using a TS-5000Z Taste Sensing System supplied by Intelligent Sensor Technology Inc., Japan [Figure 1(a)]. The fabricated taste sensor chip was connected to this taste sensing system [Figure 1(b)]. An Ag/AgCl electrode with a single ceramic junction was used for measurement of the membrane potential as a reference electrode. The measurement procedures for evaluation of the fabricated taste sensor chip and the sensor probe were performed using the same procedure, following the manual. A 30% aqueous solution of ethanol containing 100 mM hydrochloric acid was used as the washing solution for the sensor chip and for the sensor probe when testing for saltiness, sourness, and umami, while a 30% aqueous solution of ethanol containing 100 mM potassium chloride and 10 mM potassium hydroxide was used when testing for bitterness and astringency.

## 3. Results and Discussion

### 3.1. Evaluation of Sensor Chips

**Figure 3.**
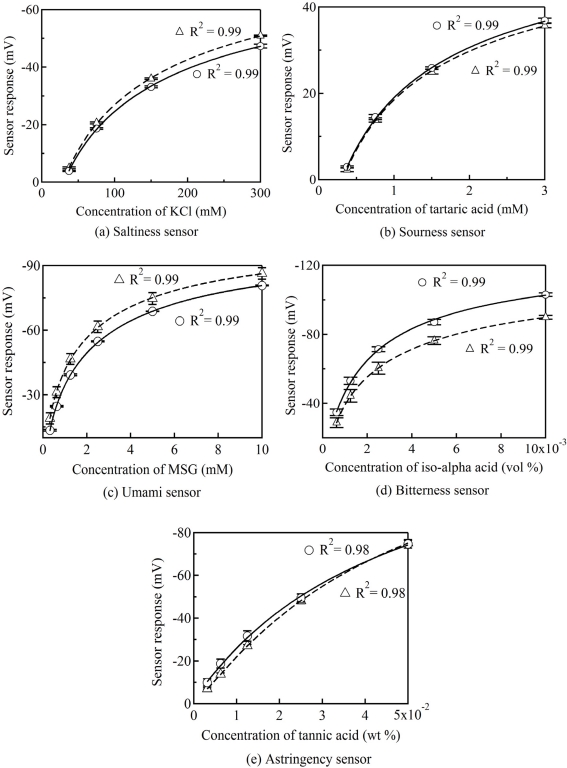
Calibration curves of the taste sensor chips and the sensor probes. Data are expressed as mean ± SD (*n* = 5). ○: sensor chip, Δ: sensor probe.

The performances of the five sensor chips for saltiness, sourness, umami, bitterness and astringency were evaluated by measuring standard taste substances. The sensor response (*V*s-*V*r) shows the difference in the membrane potential between the taste sample solution (*V*r) and the standard solution before measuring the sample solution (*V*s). Figure 3 shows the concentration characteristics of the sensor chip and the sensor probe for standard taste substances, where all of the data are expressed as the mean ± the standard deviation (SD). The calibration curves were fitted using a lognormal. With regard to calibration curves for the sensor chips and the sensor probes, the values of the coefficient of determination (R^2^) are larger than 0.97. The result demonstrates that the fabricated sensor chips have a high sensitivity for standard taste substances.

The fabricated sensor chip consisted of an electrolyte layer with KCl and an artificial lipid membrane. Polycarbonate and polyimide are well known as low-cost engineering plastics. Other studies have reported that the sensor signal of a miniaturized ion-selective chip electrode show high stability in terms of electric-potential when using pHEMA with KCl as the electrolyte layer [13]. The sensor responses for the standard taste substances showed high accuracy because of the use of pHEMA with KCl as the electrolyte layer. Additionally, it could be an advantage for commercialization of the product thanks to its simple manufacturing procedure.

### 3.2. Comparison of Sensor Chips and Sensor Probes

Linear regression analysis was used to assess the relationship between the sensor responses of the fabricated sensor chips and the conventional sensor probes (Table 2). The slopes of the regression equations for the saltiness, sourness, umami, bitterness and astringency sensors are 0.94, 1.01, 0.99, 1.12 and 0.90, respectively. Moreover, the R^2^ values are 0.99, 0.99, 0.99, 0.99, and 0.98, respectively. The results in Table 2 demonstrate that the sensor responses of the fabricated sensor chips to the standard taste substances showed high correlation with the conventional taste sensor probes. Thus, it is indicated that the fabricated taste sensor chip is capable of performing at the same level as the conventional sensor probe in a taste sensing system. The commercialized taste sensing system has applications in the manufacture of various foods. Furthermore, the sensor responses from a number of taste sensors can express multivariate taste information by generating a taste map and a radar chart [3]. The results show that a portable-type taste sensing system can be constructed by using the fabricated sensor chips as alternatives to conventional taste sensor probes.

### 3.3. Sensor Chips Repeatability

**Figure 4.**
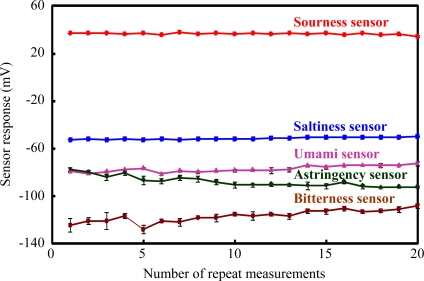
Repeatability of measurement of each taste substances by the sensor chips. Data are expressed as mean ± SD (*n* = 4). Taste substance of saltiness, sourness, umami, bitterness and astringency were used 300 mM KCl in 30 mM tartaric acid, 3 mM tannic acid in 30 mM KCl, 10 mM MSG, 0.01 vol% iso-alpha acid in reference solution and 0.05 wt% tannic acid, respectively.

The measurement repeatability for the fabricated sensor chips is summarized in Figure 4. The mean values ± SD of the sensor response to saltiness, sourness, umami, bitterness and astringency substances by the saltiness, sourness, umami, bitterness and astringency sensor chips are 51.3 ± 0.8, 36.6 ± 0.74, 77 ± 2.5, 116.4 ± 5.7 and 85 ± 3.9 mV, respectively, and the values of the coefficient variations (CVs) are 1.6, 2.0, 3.3, 4.9 and 4.6%, respectively. These results show that the sensor responses of the fabricated taste sensor chips had high stability and good reproducibility (CVs < 5%) in 20 repeated measurements. This indicated that the fabricated taste sensors for saltiness, sourness, umami, bitterness and astringency have sufficient sensor performance for the fabrication of a disposable low-cost taste sensor chip.

## 4. Conclusions

The main achievement of this study was to successfully fabricate miniaturized taste sensor chips (40 mm × 26 mm × 2.2 mm) consisted of an electrolyte layer and an artificial lipid membrane for use in a portable-type taste sensing system. The sensor responses to the standard taste substances showed high accuracy and good reproducibility. Moreover, it was revealed that the fabricated taste sensor chips displayed comparable sensor performance to a conventional sensor probe in a taste sensing system. Thus, the fabricated taste sensor chip can be used as a key element for the realization of a portable-type taste sensing system. Work on development of this kind of system is now underway.

## Figures and Tables

**Table 1.** Chemical components of the artificial lipid membranes.

**Taste sensor**	**Lipid**	**Plasticizer**
Saltiness	Tetradodecylammonium chloride *n*-Tetradecyl alcohol	Dioctyl phenylphosphonate
Sourness	Phosphoric acid di(2-ethylhexyl) ester Oleic acid Trioctylmethylammonium chloride	Dioctyl phenylphosphonate
Umami	Phosphoric acid di(2-ethylhexyl) ester Trioctylmethylammonium chloride	Dioctyl phenylphosphonate
Bitterness	Tetradodecylammonium chloride	Dioctyl phenylphosphonate
Astringency	Tetradodecylammonium chloride	2-Nitrophenyl octyl ether

**Table 2.** Linear regression analysis of the correlation between sensor responses. *Res*_chip_: sensor response of the sensor chips; *Res*_probe_: sensor response of the sensor probes.

**Taste sensor**	**Regression equations**	**R^2^**
Saltiness	*Res*_chip_ = 0.95 × *Res*_probe_ + 0.80	0.99
Sourness	*Res*_chip_ = 1.01 × *Res*_probe_ + 0.55	0.99
Umami	*Res*_chip_ = 0.99 × *Res*_probe_ + 5.97	0.99
Bitterness	*Res*_chip_ = 1.12 × *Res*_probe_ – 2.47	0.99
Astringency	*Res*_chip_ = 0.90 × *Res*_probe_ – 6.72	0.98
